# Sensational Dreams: The Prevalence of Sensory Experiences in Dreaming

**DOI:** 10.3390/brainsci14060533

**Published:** 2024-05-24

**Authors:** Anna C. van der Heijden, Jade Thevis, Jill Verhaegen, Lucia M. Talamini

**Affiliations:** 1 Department of Psychology, Brain and Cognition, University of Amsterdam, 1018 WT Amsterdam, The Netherlands; a.c.vanderheijden@uva.nl (A.C.v.d.H.); jill.kirchhof@student.uva.nl (J.V.); 2 Amsterdam Brain and Cognition, University of Amsterdam, 1001 NK Amsterdam, The Netherlands

**Keywords:** dreams, dream diary, sensory dream experiences

## Abstract

Dreaming, a widely researched aspect of sleep, often mirrors waking-life experiences. Despite the prevalence of sensory perception during wakefulness, sensory experiences in dreams remain relatively unexplored. Free recall dream reports, where individuals describe their dreams freely, may not fully capture sensory dream experiences. In this study, we developed a dream diary with direct questions about sensory dream experiences. Participants reported sensory experiences in their dreams upon awakening, over multiple days, in a home-based setting (n = 3476 diaries). Our findings show that vision was the most common sensory dream experience, followed by audition and touch. Olfaction and gustation were reported at equally low rates. Multisensory dreams were far more prevalent than unisensory dreams. Additionally, the prevalence of sensory dream experiences varied across emotionally positive and negative dreams. A positive relationship was found between on the one hand sensory richness and, on the other emotional intensity of dreams and clarity of dream recall, for both positive and negative dreams. These results underscore the variety of dream experiences and suggest a link between sensory richness, emotional content and dream recall clarity. Systematic registration of sensory dream experiences offers valuable insights into dream manifestation, aiding the understanding of sleep-related memory consolidation and other aspects of sleep-related information processing.

## 1. Introduction

Dreaming, which may be defined as a form of mental activity during sleep [1], is a long-studied phenomenon. Previous research has shown that daytime activities can shape dream content [2,3,4,5,6]. Conversely, dreaming affects daytime functioning [5,6] by enhancing memory consolidation [7,8], contributing to problem-solving [9,10] and facilitating emotional processing [11]. Thus, there appears to be a bidirectional relationship between wakefulness and dreaming.

While waking life provides a sensory rich experience, the prevalence of sensory experiences in dreams remains understudied. A few studies investigated sensory dream experiences using free recall dream reports, in which participants were instructed to freely describe their dream content [1,12,13]. These dream reports were subsequently scored for sensory experiences by an independent rater. A limitation of this approach is that participants might omit sensory dream experiences in the free recall report, obscuring the prevalence of sensory dream experiences [1,12,13].

Another study on sensory dream experiences used a single, retrospective questionnaire to assess participants’ dream experiences over the previous year [14,15,16]. This approach included direct questions on sensory dream experiences. While direct questions may help to capture the prevalence of sensory dream experiences, the long period from which dreams needed to be recalled may have led to the forgetting of dream content. Consequently, dream recall over long retention periods may again lead to distortion in the assessment of the prevalence of sensory dream experiences [14,15,16].

In this study, we employed a seven-day dream diary on sensory experiences, collected at final morning awakenings, in the home environment. By explicitly asking about sensory experiences in a dream diary, no subjective interpretation of dream content was required by an independent rater. Specifically, participants were asked to quantify the number of dreams in which they experienced vision, audition, touch, olfaction, and gustation during the previous night. By assessing dream reports upon final morning awakening, we reduced the time between the occurrence of a dream and the report, resulting in a shorter recall interval than the delayed questionnaire methods that require recalling dream details overthe past year.

We will compare the prevalence of sensory dream experiences across all sensory modalities and quantify the prevalence of combined experience of different senses in multisensory dream experiences. Prevalence will be determined as a percentage of both the total number of reports and participants; the latter to evaluate potential interindividual differences in the ability to experience each sensory modality. Additionally, the prevalence of sensory modalities will be compared between emotionally positive and negative dreams. Finally, we will explore the relationship between sensory richness and dream emotionality, as well as the relationship between sensory richness and clarity of dream recall, for both positive and negative dreams.

## 2. Materials and Methods

### 2.1. Sample and Procedure

In total, 611 participants were recruited for this study. All participants were asked to fill in the dream diary for seven consecutive days upon final morning awakening. Incomplete diary reports for any day resulted in the exclusion of only the corresponding diary entry. Participants who completed the diary on fewer than two days were excluded, resulting in a final sample of 533 participants (78.6% female, 21.4% male) with a mean age of 22.0 (SD ± 7.24, ranging from 18 to 70 years old; see Supplemental Figure S1). Participants were recruited through the participant pool of the Department of Psychology from the University of Amsterdam, various online social media platforms, and flyers. Inclusion criteria were a minimum age of 18 years old, normal or corrected-to-normal hearing and vision and no other sensory impairments. Participation was voluntarily; no financial compensation was provided. All procedures were approved by the ethical committee of the University of Amsterdam.

Participation occurred online via the questionnaire application Qualtrics (Qualtrics, Provo, UT, USA). Participants completed an entry survey to provide their contact details, age, biological sex, and country of residence. Next, participants digitally received the sensory dream diary for seven consecutive days. To aid participants, email reminders containing a link to the diary were sent each morning.

### 2.2. Sensory Dream Diary

A customized dream diary, comprising twenty-two items, was developed for this study. Participants reported the total number of dreams experienced during the previous night. If participants reported having had one or more dreams, they were next asked to indicate the presence of specific sensory experiences, such as vision, audition, touch, olfaction, and gustation, cumulatively across all reported dreams. Follow-up, open-ended questions were presented for each indicated sensory modality, asking about the number of dreams in which the sense occurred.

In addition, participants were asked to rate the emotionality of the dream eliciting the strongest positive emotion and the one evoking the strongest negative emotion using a seven-point Likert scale (0 = “none”, 6 = “extreme” [17]). Subsequently, sensory dream experiences were assessed for the most positive and negative dream separately. For all questions, an “I do not remember” or “None of the above” option was present to avoid the reporting of false positive sensory experiences.

The diary included basic questions on sleep quality and duration [18], as well as questions on the consumption of drugs and medication. It allowed multiple dreams per night to be reported. Reporting the actual dream content was optional. In case the participant reported to not recall any dreams, questions on dream content were automatically skipped. The expected completion time ranged from five to ten minutes. The complete diary has been provided in Supplemental Material S1.

### 2.3. Statistical Analyses

Jamovi 2.4.8 was used for all statistical analyses. Two-tailed tests were performed for all analyses.

#### 2.3.1. Sensory Dream Experiences

To compare the prevalence of sensory dream experiences among all sensory modalities, a generalized linear mixed model (GLMM) was employed, executed using the GAMLj3 package of Jamovi. The fixed factor was the sensory modalities (vision, audition, touch, gustation, and olfaction), and the dependent variable was the dream report count per sensory modality. The multiday diary reports per participant were included as a random effect to take into account the interindividual variability in the number of dream experiences. The following settings were applied: Poisson distribution, logit link function, the Restricted Maximum Likelihood (REML) estimation method, the random effects test Likelihood Ratio test (LRT), and a Bonferroni post hoc correction. All participants were included in this analysis, comprising 3476 dream diaries.

In addition, to obtain a measure of intersubject variability in the prevalence of sensory dream experiences, we calculated a percentage of all participants who reported this sensory experience at least once during the seven-day study period. This percentage was calculated for all sensory modalities independently.

#### 2.3.2. Multisensory Dream Experiences

To assess how different sensory components co-occur within a single dream, we selected diaries that included only one dream per night to prevent overlap across multiple dreams. This resulted in a subsample of 1221 diaries, obtained from 498 participants. For all possible (two- to five-way) combinations of the five sensory modalities, a percentage of the total dream reports was calculated.

#### 2.3.3. Link between Sensory Richness, Emotionality, and Clarity

To study a potential link between sensory richness and emotionality in dreams, reports were selected based on the presence of emotion. Exclusions were only made when emotion was indicated as not remembered; otherwise, reports were included even if emotion was reported to be absent. This resulted in a subsample of 1650 diaries from 497 participants for positive dreams and 1567 from 494 participants for negative dreams. The relationship between sensory richness and emotionality was examined separately for positive and negative emotions. Sensory richness was quantified as the percentage of sensory modalities present (e.g., presence of all five senses yielded a 100% score). Dream emotionality was assessed using a seven-point Likert scale (0 = None, 6 = Extreme). Spearman’s correlation tests were conducted including sensory richness and emotional intensity for positive and negative emotions.

In addition, a potential relationship between sensory richness and dream clarity was studied. Dream clarity denotes the extent to which dream content could be recalled upon waking and was assessed by a rating on a scale from 1 to 10 (1 = extremely unclear, 10 = extremely clear). Spearman’s correlation was applied. Additionally, a generalized linear mixed model was applied to compare dream clarity between recent and remote dreams, with dream clarity used as dependent variable, dream remoteness as fixed factor, and the participant as random factor. Recent dreams were defined as occurring within the same hour of final awakening, whereas remote dreams occurred longer than one hour before final awakening. To be able to link the dream occurrence to an individual dream, reports were selected with a total of one dream per night, in which the dream occurred before final awakening and diary entry after final awakening, resulting in a subsample of 607 reports.

Finally, to assess whether the prevalence of sensory modalities differed between positive and negative dreams, we utilized a 2 (positive and negative emotion) × 5 (sensory modalities: vision, audition, touch, gustation, olfaction) chi-square test. Here, a subsample of dreams that included emotion was selected (based on the 7-point Likert scale, emotion was ≥1). To address imbalances in the counts of different emotionality types, we employed undersampling. This involved matching the count of the most frequent emotionality type to the least frequent, resulting in a subsample of 1117 reports per emotionality type. Post hoc comparisons were conducted in R 4.3.1 [19] using the chisq.posthoc.test package, including a Bonferroni correction.

## 3. Results

### 3.1. Demographics

**Figure 1:**
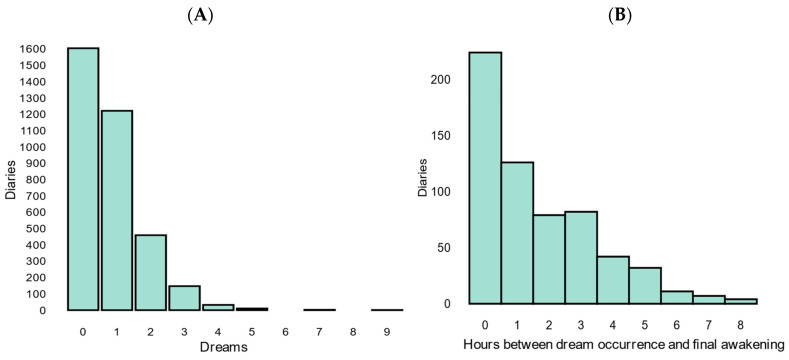
(**A**) Number of dreams per night and (**B**) hours between dream occurrence and final awakening. (**A**) The distribution of the number of dreams per night has been illustrated. Most diaries reported zero dreams, while one dream per night was the most common quantity if a dream occurred. The range of reported dreams per night spanned from zero to nine, displaying a descending pattern where higher numbers of dreams tended to occur less frequently. (**B**) The histogram displays the distribution of dream occurrences, expressed in hours before final awakening. In order to link the time of dream occurrence to a single dream, only reports with one dream per night were selected.

In total, 533 participants (78.6% female, 21.4% male) with a mean age of 22.0 (SD ± 7.24) were included, accounting for a total of 3476 reported dreams. Across all diaries, participants slept for 7 h and 38 min on average and reported a moderate-to-good sleep quality (score 3.5 out of 5). The average number of dreams per night was determined by averaging the number of dreams over all days per participant and subsequently taking the group average. Participants reported an average of 0.80 (±0.74) dreams per night. Zero dreams per night were reported most frequently, while one dream per night was the most common quantity if a dream occurred (Figure 1A). On average, participants completed the diary for 6.52 (±1.02) out of 7 days (Supplemental Figure S2). Most dreams were reported to occur within the same hour as the final awakening (Figure 1B). The average clarity of the dream recall was 5.16 (±2.32, on a scale from 1 (extremely unclear) to 10 (extremely clear). See Table 1 for all descriptives.

### 3.2. Sensory Dream Experiences

**Figure 2:**
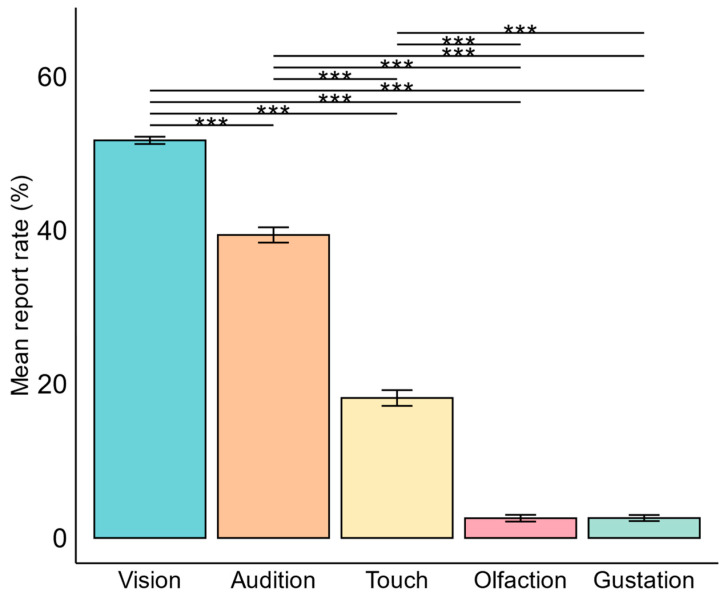
Comparison of the prevalence of sensory dream experiences. In analyzing the prevalence of sensory dream experiences, vision emerged as the most frequent sensation compared to all other senses (*p* < 0.001). Audition followed as the second most prevalent sensation, surpassing touch, olfaction, and gustation (*p* < 0.001). Touch ranked third in prevalence, exceeding olfaction, and gustation (*p* < 0.001). Olfaction and gustation exhibited equally low rates of occurrence (*p* > 0.05). The mean report rate percentage was calculated in two steps: first, the percentage of dreams containing the sensory dream experience was calculated over all reports per participant, and then this percentage was averaged across all participants. Error bars represent standard deviation. *** = *p* < 0.001.

The prevalence of sensory dream experiences was compared across sensory modalities using a generalized linear mixed model (GLMM), using sensory modality as a fixed factor, report count as a dependent variable, and participant as a random factor. The GLMM revealed a significant difference in the prevalence of different sensory modalities in dreams (χ^2^ = 2621, df = 4.00, *p* < 0.001). In the subsequent post hoc analyses with Bonferroni correction, significant differences in prevalence emerged across all senses, except for olfactory and gustatory experiences. More specific, vision was the most prevalent sensory dream experience (51.7%), followed by audition (39.4%) and touch (18.2%) (*p* < 0.001). Olfaction (2.6%) and gustation (2.6%) occurred at equally low rates (*p* > 0.05) (Figure 2).

To assess potential interindividual differences in the occurrence of sensory dream experiences, we computed, per sensory modality, the percentage of participants who reported this modality at least once during the seven-day diary period. We found that nearly all participants experienced vision (95.9%). In addition, both audition (85.6%) and touch (62.1%) were experienced by most participants. In contrast, gustation (16.5%) and olfaction (14.6%) were reported by a minority. While gustation and olfaction occurred at equal rates across all dream reports (2.6%), gustation was experienced by a numerically higher number of participants than olfaction. Moreover, amongst participants who reported dreams, 0.2% reported to not have had any sensory experiences, whereas 1.4% reported to have dreamt but to be unsure whether sensory experiences were present. The latter supports the idea that sensory experiences are paired with dream recall in most, but not all, cases. These findings demonstrate a notable intersubject variability in sensory dream experiences, highlighting that no sensory modality was experienced by all participants.

### 3.3. Multisensory Dream Experiences

**Figure 3:**
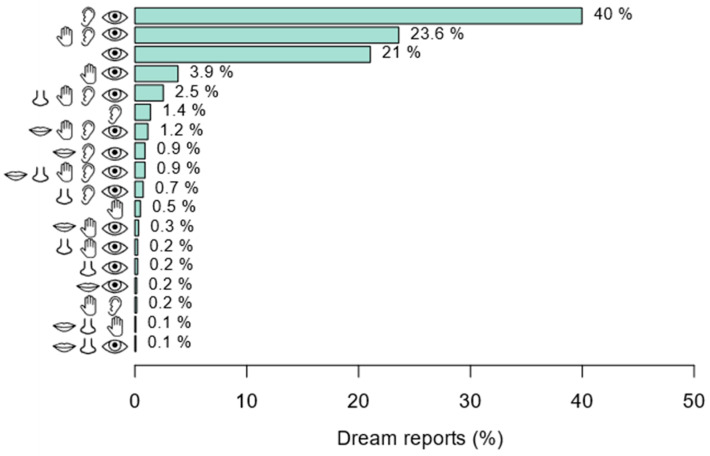
Prevalence of combinations of sensory modalities in dream experiences. This chart represents the composition and prevalence of multisensory dream experiences (prevalence is expressed as a percentage of total dream reports that contained a single dream). Sensory combinations not represented in the chart were not reported by any participant (report rate of 0%). These include the less frequently reported senses, gustation, and olfaction, occurring in isolation or in 2-, 3- and 4-way combinations with other senses. Moreover, 2.1% of dreams did not include any sensory experiences. Icons represent the following sensory dream experiences: eye = vision, ear = audition, hand = touch, nose = olfaction, mouth = gustation.

Next, we evaluated the prevalence of combinations of sensory dream experiences within a single dream. A combination of audition and vision was most frequently present (40.0%), across all unique two- to five-way combinations of sensations (Figure 3). The second most prevalent sensory dream experience was a combination of vision, audition and touch (23.6%). These combinations exceeded the independent occurrence of vision (21%), audition (1.4%), touch (0.5%), olfaction (0%), and gustation (0%), demonstrating that multisensory dream experiences are more prevalent than unisensory ones. However, not all potential sensory combinations were represented, with only 58.1% of the potential multisensory combinations being reported (see Figure 3). The occurrence of dreams involving all five senses was relatively uncommon, being present in only 0.9% of reports. The least frequent, but still reported, sensory combination was composed of vision, olfaction, and gustation (0.1%), as well as vision, olfaction, and touch (0.1%). Overall, these findings indicate that multisensory dream experiences are more prevalent than unisensory dreams, with high variability in the sensory modalities involved.

### 3.4. Sensory Rich Dreams Are Associated with Higher Emotional Intensity and Clarity

To study the relationship between dream sensory richness and emotional intensity, Spearman’s rank test was performed. Sensory richness was defined as the percentage of senses present in the dream experience. A positive relationship between sensory richness and emotional intensity was found both for dreams with positive (r = 0.367) and negative dream emotion (r = 0.465) (*p* < 0.001). This indicates that dreams with richer sensory experiences tend to have more intense emotional content.

In addition, sensory richness was positively correlated with dream clarity for both positive (r = 0.247) and negative dreams (r = 0.245) (*p* < 0.001). This suggests that dreams characterized by richer sensory experiences are reported to be more clearly remembered. Of note, the strength of this correlation was less (r = 0.247) than the correlation between emotional intensity and sensory richness (r = 0.367) (*p* < 0.001). Additionally, we found that recent dreams, which were reported to occur within one hour before final awakening, tended to be more clearly remembered than remote dreams (trend-level significance; χ^2^ = 3.78, df = 1, *p* = 0.052). While dreams were most clearly remembered when occurring within one hour before final awakening, clarity did not decrease linearly with the time elapsed since the dream (see Figure S3).

**Figure 4:**
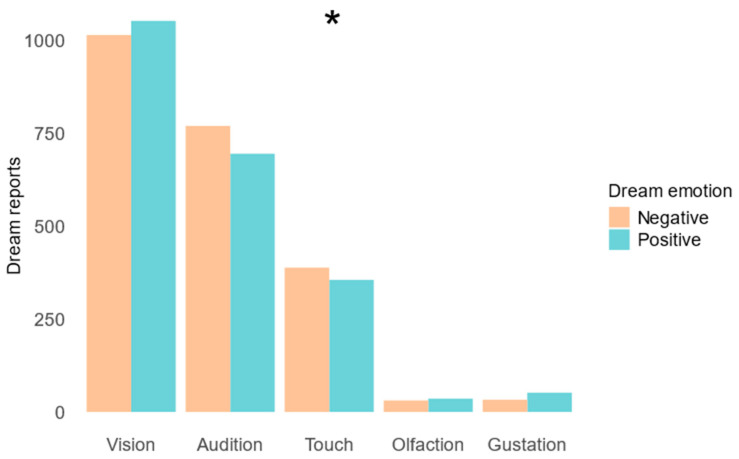
Prevalence of sensory dream experiences in emotionally positive versus negative dreams. The number of dream reports are shown for each sensory dream experience for emotionally negative (orange) and positive (blue) dreams. A chi-square test confirmed that the prevalence of sensory modalities differed amongst positive and negative dreams (χ^2^ = 10.1, *p* = 0.038). Post hoc tests comparing the prevalences of positive and negative dream reports per sensory modality did not survive correction for multiple comparisons (*p* > 0.01). * = p<0.05 of chi-square test.

Finally, we evaluated whether the prevalence of individual sensory modalities differed between dreams featuring positive and negative emotions. A chi-square test revealed that the prevalence of sensory modalities differed significantly between positive and negative dreams (χ^2^ = 10.1, *p* = 0.038). Positive dreams appeared to have a higher frequency of gustatory, olfactory, and visual sensations relative to negative dreams, whereas negative dreams seemed to exhibit more frequent auditory and tactile sensations (Figure 4). An uncorrected post hoc test demonstrated more frequent gustatory experiences in positive than negative dreams (*p* < 0.05) and a trend towards more frequent auditory experiences in negative than positive dreams (*p* = 0.05); however, these post hoc differences did not survive correction for multiple comparisons (*p* > 0.01).

In summary, sensory richness was positively associated with the intensity of dream emotion and clarity of dream recall. This relationship was present for both emotionally positive and negative dream content. In addition, sensory experiences occurred at different rates during emotionally positive and negative dreams.

## 4. Discussion

In this study, we investigated sensory dream experiences using a multiday dream diary, administered upon final morning awakenings, in a home-based setting. Our findings revealed that vision was the most prevalent sensory dream experience, followed by audition and, subsequently, touch. In contrast, olfaction and gustation occurred at similar, low rates. We observed large interindividual differences in the prevalence of sensory dream experiences. Nearly all participants reported vision (95.9%); a majority reported audition (85.6%) and touch (62.1%). Gustation (16.5%) and olfaction (14.6%) were reported by a minority. A small percentage of participants (2.1%) reported dreams in the absence of any sensory experiences. This means that during these dreams, these participants reported not experiencing any vision, sound, touch, smell, or taste. Furthermore, our results indicate that multisensory dreams were far more prevalent than unisensory dreams and that the prevalence of sensory modalities differed for emotionally positive and negative dreams. Additionally, a positive relationship was found between sensory richness and emotional intensity of dreams, both for positive and negative emotion. Similarly, sensory richness was positively associated with the clarity of dream recall, again, for both positive and negative dream emotions. These findings highlight the complexity and variability of sensory dream experiences and suggest links between sensory richness, emotional content, and clarity of dream recall.

Previous studies on this topic described a 100% report rate for the presence of visual dream experiences [1,12,20]. While vision was indeed the most prevalent sensory dream experience in our study, vision was not reported for all dreams, and some participants did not report it at all. This may indicate participants having thought like dreams without any visual imagery. It may not be possible for an independent rater to distil such dream experiences from dream narratives, resulting in a potential overestimation of visual dream experiences in previous studies [1,12,20]. Direct questions about experiencing vision in dreams, as adopted in the current study, likely improve the accuracy of classifying visual dream experiences. This approach may be particularly useful for evaluating non-rapid eye movement (NREM) dreams, which have been described as thought-like [21,22,23]. In conclusion, while sensory experiences are frequently involved in dream content, they appear not be a standard feature, even in the context of morning dreams.

While previous research overestimated visual experiences, somatosensory experiences appear to have been somewhat underestimated. Previous studies assessing sensory dream experiences in the home environment did not evaluate somatosensory experiences [12,15,16]. Studies conducted in laboratory settings, based on narrative dream reports, only showed low rates (1%) of tactile experiences [13,20]. However, our study revealed that tactile dream sensations were present in 18.2% of dream reports and experienced by a majority of participants (62.1%). This suggests a substantially higher prevalence than previously reported, again demonstrating a methodological benefit of including direct questions on sensory experiences in dream diaries. To specify the diversity of somatosensory dream experiences, follow-up studies may consider subtyping cutaneous sensations (e.g., touch, temperature, and pain) and include proprioception and kinesthesia.

In dream and memory research, a link between waking experiences and dreams has been widely acknowledged [6,8,24]. However, there seems to be a discrepancy between the prevalence of daytime and dream-related sensory experiences. While chemosensory sensations like olfaction and gustation are common during wake experience, they seem to be less frequently reported during dreaming than other sensations, like audition and vision. This applies to both our study and previous studies [2,12,25,26,27]. The putative mechanism underlying this phenomenon is uncertain. Speculatively, contributing factors may include the following: (1) less (conscious) daytime stimulation of gustation and olfaction relative to vision and audition, which reflects in sensory dream experiences; (2) infrequent replay of gustatory and olfactory memories during sleep; (3) neurobiological mechanisms in the sleeping brain that somehow inhibit chemosensory dream experiences; (4) alternatively, such dream experiences do occur but are (preferentially) forgotten in the transition to wakefulness.

Several limitations should be considered. Firstly, our study primarily captured morning dreams, as dreams occurred most frequently within the same hour as final morning awakening. An average of less than one dream per night was reported. The late dream occurrence and low report rate may indicate a lack of dream recall from earlier parts of the night [28,29]. Speculatively, given that REM (rapid eye movement) sleep is most prevalent in the second half of the night [30,31] (and dream occurrence was close to final morning awakening in our sample, this study might predominantly represent REM dreams. However, the lack of polysomnography (PSG) precluded the ability to reliably differentiate between NREM and REM dreams. Recent advancements in wearable electroencephalography (EEG) technology may facilitate sleep-monitored dream studies in the home environment and enable home-based, serial awakening paradigms [32] to distinguish NREM and REM dream content.

While self-reporting dream experiences minimizes interpretation biases by independent raters, it remains reliant on the dreamer’s evaluation, introducing potential biases. For instance, anecdotal evidence suggests instances where dreamers inferred auditory dream sensations based on visually perceiving conversations. However, upon explicit inquiry by the researcher, the participant could not confirm whether auditory dream experiences had actually been present. Addressing such nuances effectively may involve employing semi-structured interviews or providing explicit instructions in sensory dream diaries.

A final limitation concerns the composition of the sample of participants in this study, which predominantly consisted of young, female adults. Sensory dream experiences may alter with age, as sleep patterns change [33,34,35,36] and daytime sensory perception deteriorates [37,38,39,40,41,42]. Given the young sample, the findings of this study may not be generalizable to older age groups. Additionally, the sample was predominantly represented by females (78.6%). Females tend to report their dreams more frequently, as indicated by a meta-analysis based on 175 studies [43], suggesting that the dream-related prevalences reported in our study may be lower in a male population.

Future research could explore the potential link between interindividual differences in daytime sensory processing [44] and sensory experiences in dreams. Although vision dominates human perception during both waking [45] and dreaming [1,12,20], there may be subpopulations who experience certain sensory modalities more frequently than others. For instance, a previous study showed that individuals who were particularly aware of odors reported higher rates of olfactory dreams than individuals with low odor awareness [25]. Interindividual differences in sensory dream experiences that link to daytime sensory processing are further supported by evidence from studies on individuals with sensory impairments. For instance, dream experiences of the blind contain a higher prevalence of auditory and tactile sensations than those of the non-blind [46]. In deaf individuals, hearing was less frequently present compared to non-deaf dreamers, whereas gustatory, olfactory, and somatosensory dream experiences were increased compared to non-deaf dreamers [47]. Additionally, pain is more prevalent in dreams of chronic pain patients compared to healthy individuals [48]. Further investigation into sensory dream experiences involving other sensory-impaired populations may provide valuable insights into the relationship between daytime sensory experiences and dreams. Examples of such populations may consist of individuals with olfactory dysfunction, which is prevalent among long-COVID patients [49,50,51], or sensory paralysis.

## 5. Conclusions

By shifting from examining individual dream content to systematically assessing dreams through targeted inquiries about experiences (such as sensory experiences, color perception, and language), we may gain more insight into how dreams manifest. This approach could yield valuable insights into sleep-related neural processing and how this affects memory consolidation over time, further elucidating sleep’s role in strengthening and weakening memories [52,53,54,55].

## Figures and Tables

**Table 1:** Descriptives of dream reports for overall dream reports and sleep quality.

	Mean	SD
Dream diary entries	6.52	1.02
Sleep quality	3.50	0.93
Total sleep time	7:38 h	1.28 h
Bedtime	00:45 h	01:39 h
Wake-up time	08:41h	01:42 h
Number of dreams	0.80	0.74
Dream clarity	5.16	2.32
Dream occurrence (prior to final awakening)	01:39 h	1:52 h
Dream retention interval (between dream occurrence and diary entry)	02:43 h	01:39 h
Diary entry interval (between waking up and diary entry)	1:13 h	2:16 h
Number of nightmares	0.152	0.446

## Data Availability

The raw data supporting the conclusions of this article will be made available by the authors on request due to privacy reasons.

## Supplementary Materials

The following supporting information can be downloaded at: https://www.mdpi.com/article/10.3390/brainsci14060533/s1, Figure S1: Age distribution of participants in the sample; Figure S2: Quantity of diary reports per participant; Figure S3: (A) Intervals between dream occurrence and diary entry, (B) hours between waking up and diary completion; Figure S4: Distribution of dream clarity and time interval between dream occurrence and final awakening.
