# Supplementary material for: Sensational Dreams: The Prevalence of Sensory Experiences in Dreaming
Source: Brain Sci. 2024 May 24;14(6):533. doi: 10.3390/brainsci14060533 (PMC11202128; doi:10.3390/brainsci14060533)
Supplement: Supplementary file 1 [file brainsci-14-00533-s001.zip › brainsci-3001582-supplementary.pdf]

Supplemental materials S1 - Sensational Dream Diary

Question 1. **Please enter your Participant ID.**

Question 2. **Please enter the day of this dream diary record.**

Question 3. **At what time did you fall asleep last night?**

Question 4**. At what time was your final awakening?** (i.e. what was the time that you did not go back to sleep again)

Question 5. **How many hours did you sleep?**

Question 6. **How would you rate the quality of your sleep last night?**

- Very poor – Poor – Fair – Good – Very good

Question 7.1. **Which of the following substances did you use after 17:00, yesterday?** (multiple answers possible)

- Caffeine, Alcohol, Cannabis, Nicotine, Psychedelics, Sleep medication, Other substances, None of the above *(exclusive answer)*

Question 7.2. *Display this question if "Which of the following substances did you use after 17:00, yesterday? (multiple answers possible)" "Caffeine" is selected*

How many cups of coffee did you drink after 17:00?

Question 7.3. *Display this question if "Which of the following substances did you use after 17:00, yesterday? (multiple answers possible)" "Alcohol" is selected*

How many standard glasses of alcohol did you consume after 17:00?

Question 7.41. *Display this question if "Which of the following substances did you use after 17:00, yesterday?*
(multiple answers possible)" "Psychedelics" is selected
What type of psychedelics did you consume?

Question 7.42. *Display this question if "Which of the following substances did you use after 17:00, yesterday?*
(multiple answers possible)" "Psychedelics" is selected

How much did you consume?
(Estimate)

Select the corresponding unit.

- mg (milligram), g (gram), ml (milliliters), cl (centiliters)

Question 7.50. *Display this question if "Which of the following substances did you use after 17:00, yesterday?*

(multiple answers possible)" "Cannabis" is selected

What type(s) of cannabis did you consume?

- Marijuana, THC, CBD, Other

Question 7.51. *Display this question if "Which of the following substances did you use after 17:00, yesterday?*
(multiple answers possible)" "Cannabis" is selected

How much did you consume?

(Estimate)

Select the corresponding unit.

- g (gram), mg (milligram)

Question 7.6. *Display this question if "Which of the following substances did you use after 17:00, yesterday?*
(multiple answers possible)" "Nicotine" is selected

How many cigarettes did you smoke? (If you consume through vape, note that 15 puffs equals one cigarette) (Estimate)

Question 7.71. *Display this question if "Which of the following substances did you use after 17:00, yesterday?*
(multiple answers possible)" "Other substances" is selected

What "other substances" did you use after 17:00, yesterday?

Question 7.72. *Display this question if "Which of the following substances did you use after 17:00, yesterday?*
(multiple answers possible)" "Other substances" is selected

How much did you consume?
(Estimate)

Select the corresponding unit.

Please only select the options "pills" or "sprays" in case you do not know the dosage in (milli)grams/milliliters, otherwise please select the (milli)grams/milliliters option.

- mg (milligram), g (gram), ml (milliliters), cl (centiliters), Pills (amount), Sprays (amount)

Question 7.81. *Display this question if "Which of the following substances did you use after 17:00, yesterday?*
(multiple answers possible)" "Sleep medication" is selected

What type of sleep medication did you take?

Question 7.82. *Display this question if "Which of the following substances did you use after 17:00, yesterday?*
(multiple answers possible)" "Sleep medication" is selected

How much did you take? (Estimate)

Select the corresponding unit.

Please only select the options "pills" or "sprays" in case you do not know the dosage in (milli)grams/milliliters, otherwise please select the (milli)grams/milliliters option.

- mg (milligram), g (gram), ml (milliliters), cl (centiliters), Pills (amount), Sprays (amount)

Question 8**. How many dreams do you remember from last night?**

Skip to end of survey if "How many dreams do you remember from last night?" is equal to 0

Question 9. **How clearly do you remember the content of your dream(s), on a scale from 1 (extremely unclear) to 10 (extremely clear)?**

Question 10. **Which sensory components did you experience in your dream(s) last night? (multiple answers possible)**

- Vision (e.g., seeing images, colors, shades, etc.), Sound (e.g., speech, music, etc.), Smell, Touch (e.g., sensing temperature, physical pain, vibration, someone or something touching your skin, etc.), Taste, None of the above *(exclusive answer),* I do not remember *(exclusive answer)*

Question 11. *Display this question if "Which sensory components did you experience in your dream(s) last night?*
(multiple answers possible)" "Vision" is selected
In how many dreams did you experience vision? (e.g., seeing images, colors, shades)?

Question 12. *Display this question if "Which sensory components did you experience in your dream(s) last night?*
(multiple answers possible)" "Sound" is selected

In how many dreams did you experience sound? (e.g., speech, music etc.)?

Question 13. *Display this question if "Which sensory components did you experience in your dream(s) last night?*
(multiple answers possible)" "Smell" is selected

In how many dreams did you experience smell?

Question 14. *Display this question if "Which sensory components did you experience in your dream(s) last night?*
(multiple answers possible)" "Touch" is selected

In how many dreams did you experience touch (e.g., sensing temperature, physical pain, vibration, someone or something touching your skin)?

Question 15. *Display this question if "Which sensory components did you experience in your dream(s) last night?*
(multiple answers possible)" "Taste" is selected

In how many dreams did you experience taste?

Question 16. **How many of your dreams last night could be classified as a nightmare?** (Nightmare definition: a dream with a strong negative emotional tone after which you woke up immediately)
(I do not remember = -1, none = 0)

Question 17. **Please think back to the dream with the strongest positive emotion(s) (e.g. happiness, enthusiasm, proudness, love). What was the intensity of the positive emotion in this dream?**

Intensity positive emotion

I do not remember *(-1)*	None *(0)*	Slight *(1)*	Somewhat *(2)*	Moderate *(3)*	Quite a bit *(4)*	Very much *(5)*	Extreme *(6)*

Question 18. *Display this question if "Please think back to the dream with the strongest positive emotion(s) (e.g. happiness, enthusiasm, proudness, love). What was the intensity of the positive emotion in this dream?" is greater than or equal to 0*

Which sensory components were present in this dream - in which you experienced the strongest positive emotion(s)?
(multiple answers possible)

- Vision, Sound, Smell, Touch, Taste, None of the above *(exclusive answer),* I do not remember *(exclusive answer)*

Question 19. **Please think back to the dream with the strongest negative emotion(s) (e.g. anger, sadness, fear). What was the intensity of the negative emotion in this dream?**

Intensity negative emotion

I do not remember *(-1)*	None *(0)*	Slight *(1)*	Somewhat *(2)*	Moderate *(3)*	Quite a bit *(4)*	Very much *(5)*	Extreme *(6)*

Question 20. *Display this question if "Please think back to the dream with the strongest negative emotion(s) (e.g. anger, sadness, fear). What was the intensity of the negative emotion in this dream?" is greater than or equal to 0*

Which sensory components were present in this dream - in which you experienced the strongest negative emotion(s)? (multiple answers possible)

- Vision, Sound, Smell, Touch, Taste, None of the above *(exclusive answer),*I do not remember *(exclusive answer)*

Question 21. **Please give an estimation of when your dreams occurred during the night.** (multiple answers possible)

- Before 19:00, 19:00 – 20:00. 20:00 – 21:00, (…), 11:00 – 12:00, 12:00 – 13:00, After 13:00, I do not remember *(exclusive answer)*

Question 22. OPTIONAL:
Would you like to report the specific content of your dreams from last night?

Supplemental results

S1 – Age distribution in sample

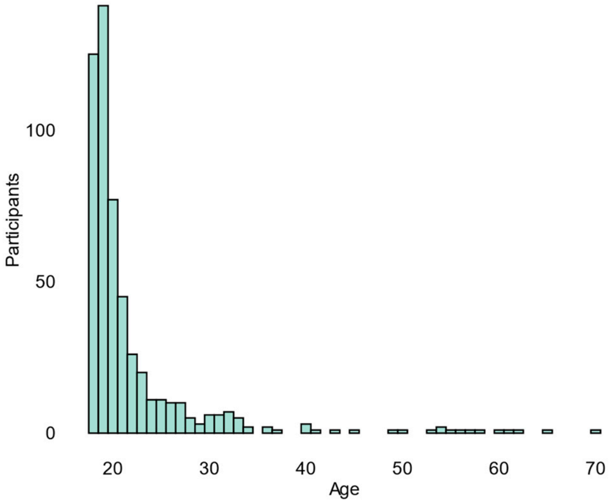

Figure S1 – Age distribution of participants in the sample

The age distribution of participants in this sample ranged from 18 to 70 years old. Young adults between 18-35 years old were most frequently represented.

S2 – **Quantity of diary reports per participant**

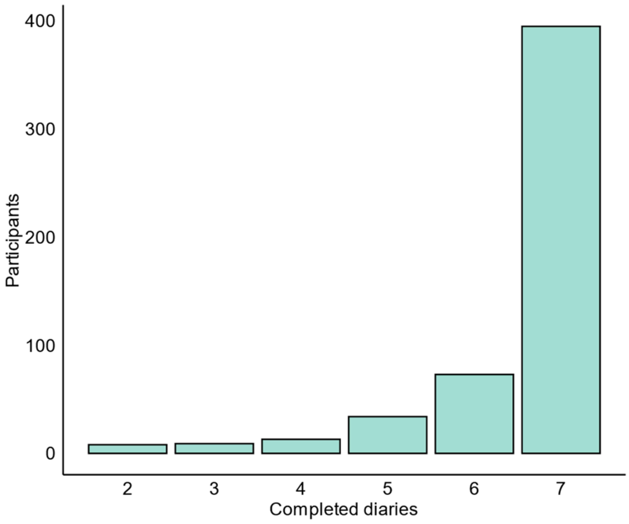

Figure S2 – Quantity of diary reports per participant

The majority of participants completed seven out of seven diaries. All participants completed at least two or more diaries.

S3 – Time interval disributions of dream retention

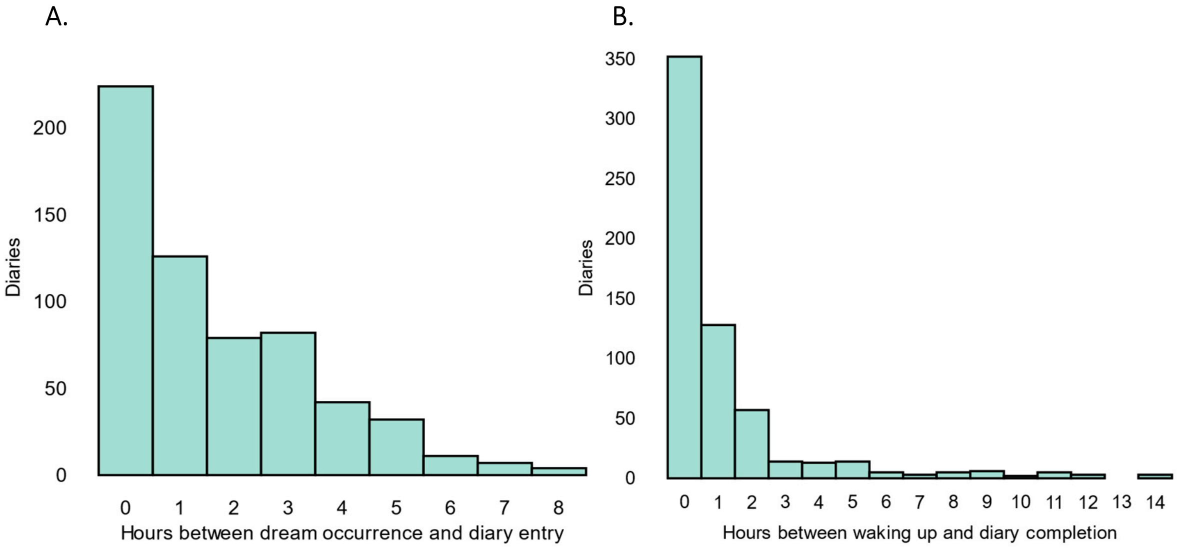

Figure S3 – S3A Intervals between dream occurrence and diary entry, S3B hours between waking up and diary completion

Most dreams occurred within the same hour as when the diary was filled in (S3A). Most diaries were completed within the same hour after waking up (S3B), indicating a short time spent awake before completing the diary. In order to link the time of dream occurrence to a single dream, only reports with one dream per night were selected.

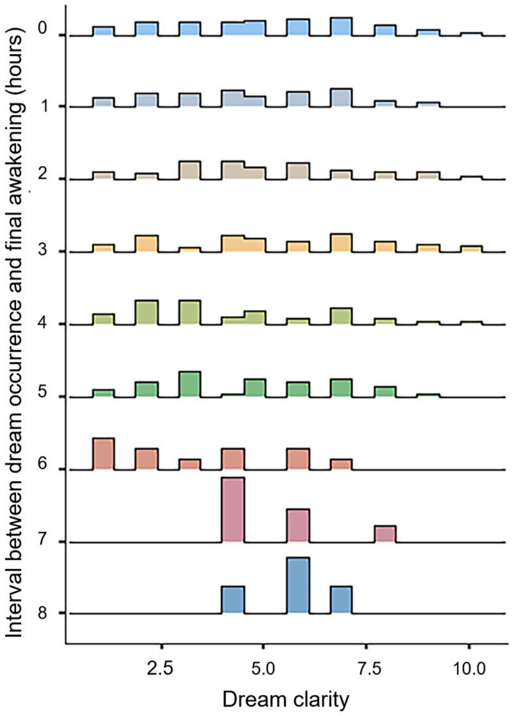

Figure S4 – Distribution of dream clarity and time interval between dream occurrence and final awakening.

The graph shows the distribution of dream clarity over recent and remote dreams, categorized by different time intervals of when the dream occurred prior to final awakening. Dreams were most clearly remembered within one hour before final awakening, however, clarity did not decrease linearly with the time elapsed since the dream. Specifically, the most remote dreams were not necessarily the least clearly remembered. Note: Dream clarity indicates how clearly the dream was remembered, with higher scores representing higher clarity.
